# Iron Deficiency in Heart Failure: What Do We Know So Far?

**DOI:** 10.7759/cureus.30348

**Published:** 2022-10-16

**Authors:** Amr Elkammash, Rasha M Farahat, Aya Al Sattouf, Julius Lenaerts, Khin Yadanar Maung, Aayesha Khatri

**Affiliations:** 1 Cardiology, Royal Papworth Hospital NHS Foundation Trust, Cambridge, GBR; 2 Medicine, West Suffolk NHS Foundation Trust, Suffolk, GBR; 3 Internal Medicine, West Suffolk NHS Foundation Trust, Suffolk, GBR; 4 Intensive Care Unit, King’s College NHS Foundation Trust, London, GBR

**Keywords:** metabolism, anemia, heart failure with reduced ejection fraction, heart failure with preserved ejection fraction (hfpef), deficiency, ferric carboxymaltose, heart failure, iron

## Abstract

Iron is vital for multiple biological processes in the human body. Heart failure (HF) patients are at a high risk of becoming iron deficient. Iron deficiency is a marker of severe HF and an ominous sign of poor outcomes. Iron deficiency can be absolute (low iron stores) or functional (improper functioning in the metabolic processes). The European Society of Cardiology recommends routine screening of iron stores in HF patients using ferritin and transferrin saturation. It advises iron replacement in deficient patients irrespective of the presence of anemia. Iron replacement improved HF symptoms, exercise capacity, and quality of life in deficient patients. It alleviates their disordered breathing during sleep. Therefore, the treatment of iron deficiency is an important target in managing HF. Oral iron is not effective in repleting iron stores in HF patients. Intravenous iron is an effective way to replenish iron stores in this cohort.

## Introduction and background

**Figure 1:**
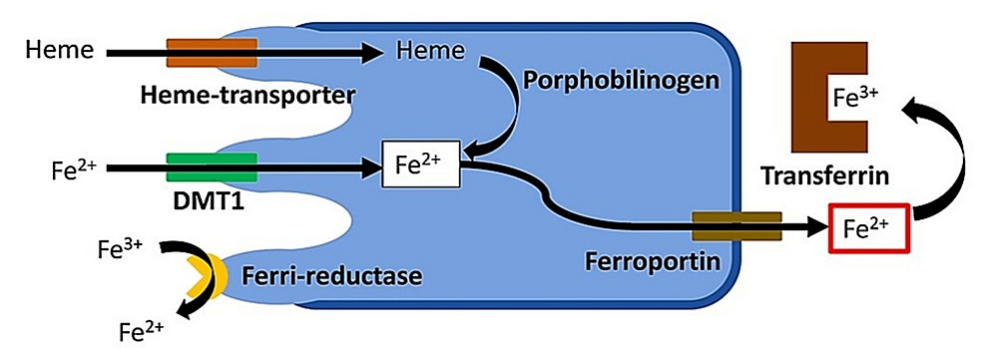
Iron metabolism at the cellular level. The figure is created by the author Julius Lenaerts. Adapted from von Haehling et al. [5].

Iron is a necessary component of all life forms because it can take and give electrons to change from its ferrous form to its ferric form [1]. Iron in the human body contributes to the function of skeletal muscle, the thyroid gland, the central nervous system, and the immunological system, in addition to the transfer of oxygen. About 3-5 g of iron are present in a single individual, of which two-thirds are found inside hemoglobin [2]. In certain cell types, stored iron linked to ferritin plays a significantly lower role: 800 to 1,000 mg in men and 300 to 500 mg in women. This helps to explain why female patients have a higher prevalence of iron deficiency (ID). The body always prioritizes the utilization of iron for metabolic processes, with erythropoiesis having a relative advantage over other processes in terms of function [3]. Iron also works as a cofactor for enzymes or a component of proteins with specific cellular functions [4]. Figure 1 illustrates iron metabolism in the body.

**Figure 2:**
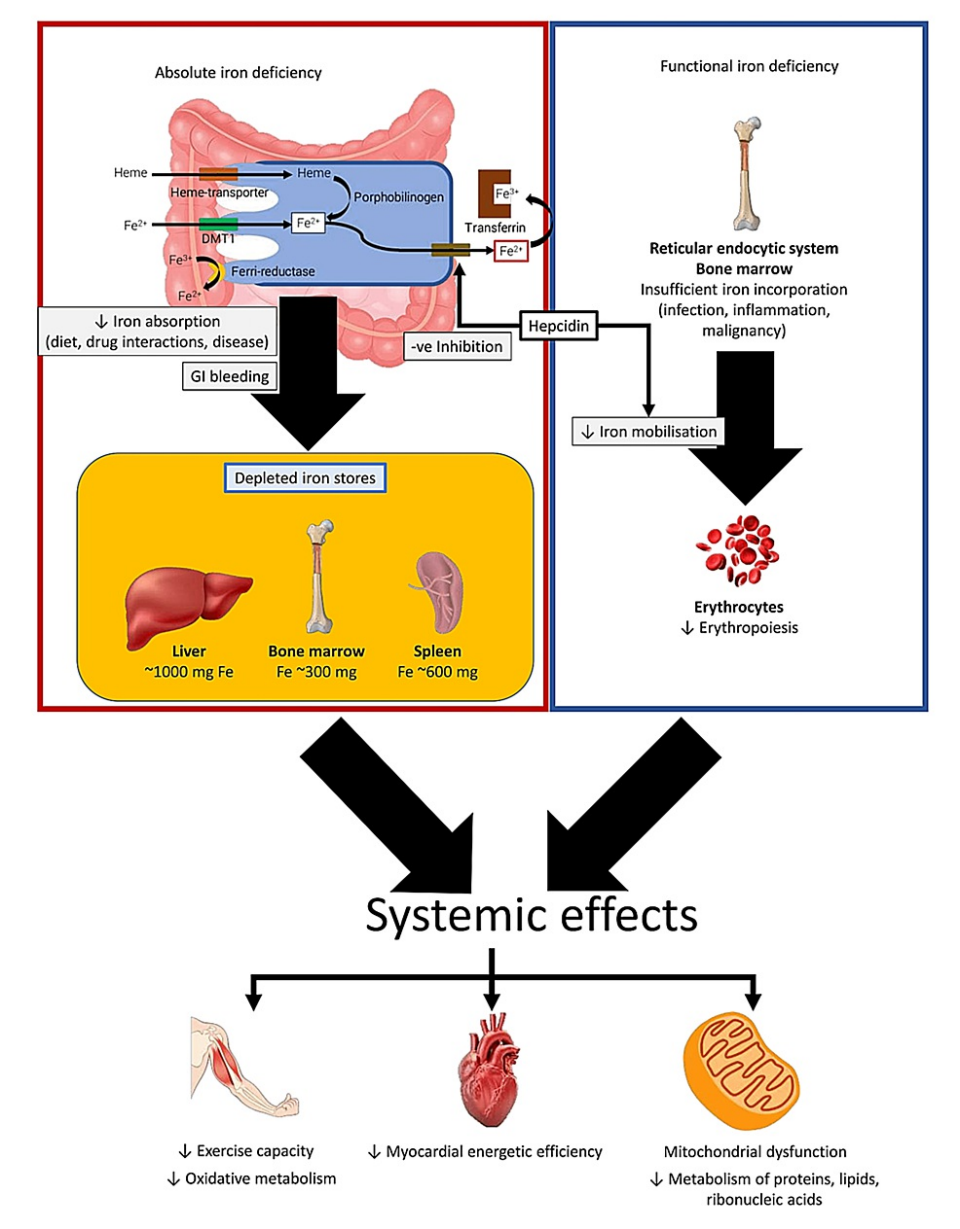
Types of iron deficiency and its effects on the body systems. The figure is created by the author Julius Lenaerts. Adapted from Loncar et al. [10].

Iron deficiency (ID) is a clinical condition that occurs when the body's requirements for iron are not met [6]. Two types of ID are distinguished for clinical and didactic purposes: absolute and functional. Depleted iron stores are the hallmark of absolute ID, even though erythropoiesis, regulatory mechanisms, and iron transport are unaffected [7]. Contrarily, functional ID denotes a mismatch between tissue supply and demand for iron, mainly as a result of iron maldistribution and iron use [6]. ID causes mitochondrial dysfunction and increases the oxidative load in the myocardial cells [8], leading to impairment of the myocardial energetics and function [9] (Figure 2). In this article, the authors demonstrate the latest available literature on ID in heart failure (HF) patients to improve the management of this vulnerable group.

## Review

Diagnosis of iron deficiency in heart failure patients

**Figure 3:**
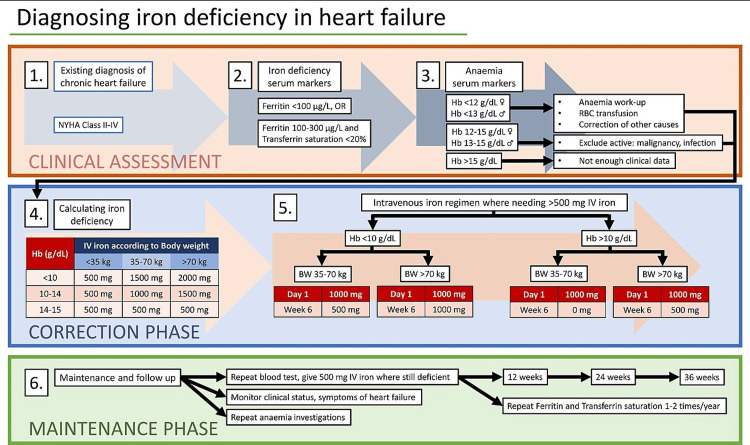
Diagnosis, treatment, and follow-up of iron deficiency in heart failure patients. The figure is created by the author Julius Lenaerts. Adapted from Loncar et al. [10].

The European Society of Cardiology (ESC) guidelines have established serum ferritin levels of 100 µg/L or ferritin levels of 100 to 300 µg/L with transferrin saturation (TSAT) of 20% as cut-off values for detecting iron shortage in HF [11] (Figure 2). The challenge of appropriately diagnosing ID results from the contrast between mobilizable and immobilizable iron and between stored and circulating iron. The patient's condition is crucial in determining if functional ID develops, as chronic heart failure (CHF), like chronic renal disease, inflammatory bowel disease, or cancer, has been linked to increased systemic inflammation [6]. Since 2012, the ESC recommendations have advised that all HF patients be tested for ID utilizing serum ferritin and TSAT assessments. Recommendations per the 2021 ESC guidelines and recent trial findings suggest that clinicians should routinely assess ID and anemia in all patients with HF as part of the clinical evaluation (i.e., once or twice a year, depending on the severity of the ID and the HF). Additionally, individuals with suspected CHF, ambulatory patients with deteriorating HF, and those who have undergone hospitalization for acute heart failure (AHF) should have their iron status evaluated [5] (Figure 3).

Prevalence of iron deficiency in heart failure patients

About one-third of the general population suffers from ID, one of the most prevalent nutritional deficiencies in the world [12]. Regardless of gender, race, or left ventricular ejection function, ID is a primary cause of anemia in patients with stable CHF, with a 30-50% prevalence [13,14]. In addition, more than 40% of CHF patients who do not have anemia or abnormal hematological indices have laboratory abnormalities indicating low iron storage. Additionally, up to 80% of examined patients can have ID in decompensated AHF [15,16].

The correlation between heart failure, exercise tolerance, and quality of life

ID is linked to worse HF symptoms, exercise tolerance (ET), and quality of life (QoL), with increased HF hospitalization and mortality in patients with CHF [17] (Figure 2). This effect is consistent among the different subclasses of HF: HF with reduced (HFrEF), mildly reduced (HFmrEF), and preserved (HFpEF) left ventricular ejection fraction (LVEF) [18].

ID is also considered a clinical marker of severity in patients with AHF and is linked to a more extended hospital stay and higher mortality [19].

Estimation of iron deficit

Generally, the Ganzoni formula [20] has been used to calculate the iron shortage in individuals with chronic illness. This formula assumes that the optimal hemoglobin level (Hb) is 15.0 g/dL (body weight greater than 35 kg) or 13.0 g/dL (lower body weight). Ganzoni formula does not account for replenishing iron reserves; hence, a 500 mg additional depot dose is typically added as follows:

Body weight in kg × (target Hb - actual Hb) × 2.4 + depot iron) = iron defect (mg).

Iron deficiency in acute heart failure

Ponikowiski and colleagues [21] studied 471 patients with AHF with reduced ejection fraction (i.e.HFrEF) and ID in the AFFIRM AHF trial. A total of 222 patients received IV ferric carboxymaltose (FCM), and 249 received the placebo. The mean follow-up duration was 52 weeks. The study showed that IV iron therapy was safe and improved recovery. It also massively reduced the risk of HF hospitalizations. On the contrary, Borreda and colleagues [22] showed that IV sodium ferric gluconate complex administration in AHF patients did not decrease the AHF readmission rate in a retrospective analysis.

Iron deficiency in chronic heart failure

The FAIR-HF study [23], conducted in 2009 on 459 patients, is one of the earliest studies examining iron therapy’s beneficial effects on patients with ID. In this randomized, double-blinded study, CHF patients were given FCM and compared with a placebo group. Interestingly, there was much improvement in symptoms in the group receiving the FCM; this improvement is more visibly observed in patients with HFrEF. Moreover, this study showed that iron therapy is beneficial and should be given to iron-deficient patients with or without anemia. These findings are closely mirrored by other independent studies, such as CONFIRM-HF [24] and EFFECT-HF [25].

Treatment of iron deficiency in heart failure patients

Oral Iron

There are many pitfalls to oral iron therapy, including poor absorption (particularly in patients with diminished intestinal absorption), dosage regime (which requires an empty stomach), and time to repletion of iron stores (two to six months). Almost half of the patients taking oral iron also suffer from gastrointestinal side effects; the most commonly reported are abdominal pain, flatulence, nausea, diarrhea or constipation, and black discoloration of feces [5]. Lewis and coworkers [26] showed that oral iron replacement was ineffective in improving the primary endpoint; peak oxygen uptake, or the secondary endpoints; the six-minute walk test, and N-terminal-prohormone brain natriuretic peptide (NT-proBNP) results at 16 weeks of follow-up. They noted a minimal rise in ferritin and TSAT. Therefore, oral iron replacement is not recommended in iron-deficient HF patients [5].

Intravenous Iron

IV iron replacement has remained a favorable option for ID management in HF. This is because of several factors, including swift repletion of iron stores, an excellent safety profile, and marked improvement in primary endpoints [10]. IV iron replacement also improves central chemoreceptor sensitivity, ventilatory function, and sleep-related breathing disorders in HF patients with iron deficiency anemia (IDA) [27]. The FERRIC-HF trial showed that IV iron sucrose effectively reduced HF symptoms in iron-deficient patients [28]. Da-Silva et al. [29] compared IV iron sucrose to oral ferrous sulfate and placebo in the IRON-HF trial. The study showed that IV iron sucrose effectively improved the maximum aerobic capacity in HF patients with IDA. Such an outcome was not achieved with the oral iron or the placebo.

IV FCM administration also improved the HF symptoms, six-minute walking distance, and QoL in HF patients with ID, independently of the presence of anemia [5]. The AFFIRM-HF [21], the CONFIRM-HF [24], and the FAIR-HF [23] trials have reflected these benefits. IV iron replacement also reduced the HF readmission rate, with a five-year saving of €0.8 million [30].

Table 1 summarizes the trials that studied ID management in HF patients.

**Table 1:** Summary of the trials studying the treatment of iron deficiency in heart failure patients. LVEF: left ventricular ejection fraction; Hb: hemoglobin; NYHA: New York Heart Association; TSAT: transferrin saturation; NT-proBNP: N-terminal-prohormone brain natriuretic peptide; FCM: ferric carboxymaltose; CRP: C-reactive protein; HF: heart failure

Formulation of iron replacement	Study name	ClinicalTrial.gov reference	Diagnosis	Number of patients	Randomization	Blinding	Symptoms	LVEF	Definition of iron deficiency	Hb	Natriuretic peptides	Duration	Treatment regimen	Primary endpoint
Iron sucrose	FERRIC-HF [28]	NCT00125996	HFrEF	35	2:1 (iron:placebo)	Double-blind	NYHA functional Class II–III	45%	Serum ferritin <100 ng/mL, or serum ferritin 100–299 ng/mL with TSAT <20%	<12.5 g/dL anemic Group, 12.5–14.5 g/dL non-anemia group	Not included	16 weeks	Iron sucrose 200 mg weekly until ferritin >500 ng/mL	Change in absolute pVO_2_ (mL/minute) from baseline to week 18
	Toblli et al. [31]		HFrEF	40	1:1 (iron:placebo)	Double-blind	NYHA functional Class II–IV	<35%	TSAT <20%, ferritin <100 ng/mL	<12.5 g/dL	NT-proBNP	25 weeks	Iron sucrose 200 mg weekly for five weeks	Change in the NT-proBNP level and inflammatory status by CRP
FCM	FAIR-HF [23]	NCT00520780	HFrEF	459	2:1 (FCM:placebo)	Double-blind	NYHA functional Class II–III	<40% in NYHA functional class I, <45% in NYHA functional class III	Serum ferritin <100 ng/mL, or serum ferritin 100–299 ng/mL with TSAT <20%	9–13.5 g/dL	Not included	24 weeks	200 mg FCM	Change in self-reported PGA score and NHYA Class from baseline to week 24
	CONFIRM-HF [24]	NCT01453608	HFrEF	304	1:1 (FCM:placebo)	Double-blind	NYHA functional Class II–III	<45%	Serum ferritin <100 ng/mL, or serum ferritin 100–299 ng/mL with TSAT <20%	<15 g/dl	BNP >100 pg/mL, NT-proBNP >400 pg/mL	52 weeks	200 mg FCM and 500 mg FCM	Change in six-minute walk distance from baseline to week 24
	EFFECT-HF [25]	NCT01394562	HFrEF	174	1:1 (FCM:standard of care)	Open-label	NYHA functional Class II–III	<45%	Serum ferritin <100 ng/mL, or serum ferritin 100–299 ng/mL with TSAT <20%	<15 g/dl	BNP >100 pg/mL, NT-proBNP >400 pg/mL	24 weeks	500 mg FCM three times	Change in peak VO_2_ from baseline to week 24
	FAIR-HFpEF [32]	NCT03074591	HFpEF	200	1:1 (FCM:placebo)	Double-blind	NYHA functional Class II–III	<45% together with evidence of diastolic dysfunction	Serum ferritin <100 ng/mL, or serum ferritin 100–299 ng/mL with TSAT <20%	9–14 g/dl	BNP >100 pg/mL, NT-proBNP >400 pg/mL, MR-proANP>125 pmol/L	52 weeks	500–2,000 mg FCM according to Hb and weight value	Change in six-minute walk distance from the baseline to week 24
	FAIR-HF 2 [33]	NCT03036462	HFrEF	1,200	1:1 (FCM:placebo)	Double-blind	NYHA functional Class II–III	<45%	Serum ferritin <100 ng/mL, or serum ferritin 100–299 ng/mL with TSAT <20%	9.5–14 g/dL	Not included	Event-driven	500–2,000 mg FCM according to Hb and weight value	Combined rate of recurrent hospitalizations for HF and of cardiovascular death from baseline to at least 12 months of follow-up
	AFFIRM-AHF [21]	NCT02937454	AHF after restabilization	1,100	1:1 (FCM:placebo)	Double-blind	NYHA functional Class II–III	<50%	Serum ferritin <100 ng/mL, or serum ferritin 100–299 ng/mL with TSAT <20%	8–15 g/dl	Not included	52 weeks	500–1,500 mg FCM according to Hb and weight value	HF hospitalizations and cardiovascular death up to 52 weeks after randomization
	HEART-FID [34]	NCT03037931	HFrEF	3,014	1:1 (FCM:placebo)	Double-blind	NYHA functional Class III–IV	<35%	Serum ferritin <100 ng/mL or 100–300 ng/mL with TSAT <20%	9.0–13.5 g/dL (women) or <15.0 g/dL (men)	NT-proBNP >600 pg/mL or BNP >200 pg/mL for patients with normal sinus rhythm or NT-proBNP >1,000 pg/mL (or BNP >400 pg/mL) for patients with atrial fibrillation	Event driven	500–2,000 mg FCM according to Hb and weight value	Incidence of death and incidence of hospitalization for HF at least 12 months of follow-up and change in six-minute walk distance after six months
		NCT01978028 [35]	HFrEF	20	1:1 (FCM:placebo)	Double-blind	NYHA functional Class II–III	<40%	Serum ferritin <100 ng/mL, or serum ferritin 100–299 ng/mL with TSAT <20%	9.5–13.5 g/dL	Not included	12 weeks	500–2,000 mg FCM according to Hb and weight value	Evaluate the effect of FCM on mitochondrial gene activation pattern after 12 weeks of treatment
		NCT03218384 [36]	HFrEF	32	1:1 (FCM:placebo)	Double-blind	NYHA functional Class II–III	<40% or <40% with left atrial volume index >28 mL/m^2^	Serum ferritin <100 ng/mL, or serum ferritin 100–299 ng/mL with TSAT <20%	<13 g/dL for men and <12 g/dl for women	Not included	Four weeks	750 mg FCM	Change in skeletal muscle mitochondrial oxidative capacity
Iron isomaltoside	IRONMAN [37]	NCT02642562	HFrEF	1,300	1:1 (iron:placebo)	Open-label	NYHA functional Class II–IV	<45%	TSAT <20% and/or ferritin <100 mg/L	>9.0 and <13.5 g/dL (women) or <15.0 g/dL (men)	NT-proBNP >250 ng/L in sinus rhythm or >1,000 ng/L in atrial fibrillation, or BNP of >75 pg/mL or 300 pg/mL, respectively	120 weeks	Iron (iii) isomaltoside 1,000	CV mortality or hospitalization for worsening heart failure
Oral	IRON5 [38]	NCT02998697	HF	54	1:1 (iron:placebo)	Double-blind	NYHA functional Class II–III	<50%	Serum ferritin <100 ng/mL or 100–300 ng/mL with TSAT <20%	>8 and <13 g/dL for men and <12 g/dL for women	NT-proBNP >4,000 pg/mL	90 days	Ferrous sulfate 200 mg three times daily for 90 days	Change in six-miute walk distance from baseline to 90 days
	IRONOUT [26]	NCT02188784	HFrEF	225	1:1 (FCM:placebo)	Double-blind	NYHA functional Class II–IV	<40%	Serum ferritin <100 ng/mL, or serum ferritin 100–299 ng/mL with TSAT <20%	9.0–13.5 g/dL	NT-proBNP	16 week	150 mg oral iron polysaccharide iron complex (feramax) twice daily	Change in peak VO_2 _from baseline to week 16

## Conclusions

ID has a negative impact on HF patients in both acute and chronic settings, independent of the presence of anemia. Iron stores should be regularly checked in HF patients and replaced accordingly. Oral iron is not effective in treating ID in HF patients. IV iron effectively reduces HF symptoms and readmission rates and improves functional capacity and QoL in HF patients.
